# Biomarker for Spinal Muscular Atrophy: Expression of SMN in Peripheral Blood of SMA Patients and Healthy Controls

**DOI:** 10.1371/journal.pone.0139950

**Published:** 2015-10-15

**Authors:** Christian Czech, Wakana Tang, Teodorica Bugawan, Calvin Mano, Carsten Horn, Victor Alejandro Iglesias, Stefanie Fröhner, Phillip G. Zaworski, Sergey Paushkin, Karen Chen, Thomas Kremer

**Affiliations:** 1 Roche Pharmaceutical Research & Early Development, Neuroscience, Roche Innovation Center Basel F. Hoffmann –La Roche, Basel; 2 SMA Foundation, New York, NY, United States of America; 3 Research - Genomics & Oncology, Roche Molecular Systems, Inc., Pleasanton, CA, United States of America; 4 Roche Diagnostics GmbH, Penzberg, Germany; 5 PharmOptima Inc., Portage, Michigan, United States of America; University of Edinburgh, UNITED KINGDOM

## Abstract

Spinal muscular atrophy is caused by a functional deletion of SMN1 on Chromosome 5, which leads to a progressive loss of motor function in affected patients. SMA patients have at least one copy of a similar gene, SMN2, which produces functional SMN protein, although in reduced quantities. The severity of SMA is variable, partially due to differences in SMN2 copy numbers. Here, we report the results of a biomarker study characterizing SMA patients of varying disease severity. SMN copy number, mRNA and Protein levels in whole blood of patients were measured and compared against a cohort of healthy controls. The results show differential regulation of expression of SMN2 in peripheral blood between patients and healthy subjects.

## Introduction

Spinal muscular atrophy (SMA) is the number one genetic cause of death in infants and toddlers. It is caused by a homozygous absence or loss of function mutation of the *SMN1* gene on Chromosome 5 (5q11.2-13-3) [1] leading to a progressive loss of anterior alpha motor neurons causing weakness and atrophy of proximal muscles (for review see [2]). All SMA patients have at least one copy of SMN2, an inverted gene duplication of SMN1 that is exclusively present in humans ([3]). However, SMN2 has a transcriptionally silent C to T mutation in exon 7. This mutation drastically modifies splicing of exon 7 resulting in a high percentage of truncated SMN2 transcripts, which lacks exon 7 and is translated into an unstable protein ([4]; [5]). The SMN protein is 294-amino acids in length and expressed ubiquitously in the human body. SMN has multiple functions; it is part of a large molecular complex with a proven role in assembly of small nuclear ribonucleoproteins (snRNPs) and mRNA splicing [6] and has also been implicated in axonal transport and growth [7]). The exact function of SMN in motor neurons and the selective vulnerability of lower motor neurons to SMN deficiency are not yet fully understood.

The amount of SMN2 full length transcript, and thus of functional protein, has a potentially strong influence on the disease phenotype, which depends upon the copy number and also the expression levels of the SMN2 gene in particular in disease tissue ([8]; [9], [10]. The clinical phenotype is historically classified into four types differing by severity and age of onset. The most severe is Type 1, where the disease manifests within the first six months of life and patients are never able to sit independently and often do not live past two years of age. Type 2 patients have a disease onset between 6 and 18 months of age and acquire the ability to sit independently but never walk. Type 2 patients have reduced life expectancy but frequently live into adulthood. Type 3 patients have an onset after 18 months of age, achieve the ability to walk unaided while exhibiting significant muscle weakness and a walking disability, and have a normal life expectancy. Type 4 is the mildest form of SMA with an onset in the fourth to sixth decade of life and a normal life expectancy. It is generally accepted that rather than a disease with discrete types, SMA spans as a continuum of severity [2], and as we show below there is a clear relationship between the clinical phenotype and the copy number of the SMN2 gene.

There is currently no treatment for SMA; patient care is focused on improving quality of life by treatment of co-morbidities. Disease modifying therapeutic approaches are currently aiming to increase SMN protein levels by modifying SMN2 splicing, using gene therapy to add an exogenous copy of SMN, or protecting motor neurons from degenerating ([11] [12] for review). Recently, small molecules that modify the splicing of SMN2 toward the inclusion of Exon 7 have been published and show clear efficacy in SMA mouse models ([13]). This approach has the potential to improve the phenotype or halt the progression of the disease. The putative target tissue of SMA treatment is the spinal cord, a tissue that cannot be sampled directly to evaluate drug effects. Thus, it is important to develop meaningful and reliable measurements from accessible tissues, such as peripheral blood. Moreover, the variability in SMA phenotypes requires careful consideration of which patients are best to enroll in studies in order to be able to follow disease progression and measure response to therapy; critical to this process is the development of biomarkers that can help predict and measure responses to drugs in SMA patients using simple blood specimens. “Biomarker” is a characteristic that is objectively measured and evaluated, and that is an indicator of a biologic process. The potential advantages of biomarkers for SMA are manifold and can be applied to new drugs that are tested in this field. Here, we report on the analysis of peripheral blood SMN mRNA and protein levels and their correlation with SMN2 copy number and disease severity in order to assess their utility as biomarkers for SMA. The data show that the regulation of SMN2 expression in SMA patient blood is different from healthy controls in this sample set. We envisage that robust analysis of SMN-related biomarker readouts will be an important tool for therapeutic development and will help to more efficiently assign patients into studies, characterize their SMN expression pattern and assess their profile in disease-modifying therapeutic approaches.

## Material and Methods

### Participants and ethics

The patients for this study were recruited at the University of Utah in Salt Lake City, Utah, USA, and the Jasper Clinic in Kalamazoo, Michigan, USA, during August 14, 2013 and February 14, 2014. The protocol was approved by the local ethics committees: University of Utah IRB, 75 South 2000 East, Salt Lake City, UT 84112, Integreview IRB, 3001 S. Lamar Blvd, Suite 210, Austin, TX, 78704. The study has been registered at clinicaltrial.gov (NCT01910168) and was sponsored by Roche. All procedures were conducted according to the principles expressed in the Declaration of Helsinki. Written informed consent for participation was obtained from the subjects or their legal guardians and assent for participation was obtained directly from subjects between 7 and 17 years of age and approved by the individual institutional review boards at the participating sites. No animal data are in the study. Details on demographic and clinical characteristics are given in Table 1. Healthy controls in this study were randomly selected subjects donating blood at the blood bank of Basel (Blutspendezentrum beider Basel), Switzerland and criteria for blood donation and accreditations are outlined in detail on the web site (www.blutspende-basel.ch). Briefly, donors need to be between 18 and 60 years old, at least 50kg in body weight, not having current medical treatment, no surgery within the last 12 months and blood laboratory values within normal range. The subjects were all at least 18 years old and consented to the study. These samples were collected anonymously.

**Table 1:** Demographics of Enrolled SMA Patients.

SMA Type	n	Mean Age(years)	Min, Max Age (years)	Sex (M/F)	Mean *SMN2* Copy Number*
1	7	2.7	0.5, 9	4/3	2
2	14	10.8	0.7, 57	7/7	3.1
3	15	20.9	2, 61	7/8	3.5

*SMN2 copy number as reported by the patient and/or measured during the study. Copy number could not be determined for every patient.

### Study procedures and criteria for inclusion/exclusion of SMA patients

All patients went to the centers for a single visit. The study protocol containing detailed study procedures, inclusion and exclusion criteria and the flow diagram of patients is shown in S2 Text. In brief, blood was collected using standard procedures. Sampling was done for RNA in PaxGene tubes (Becton-Dickinson), for protein in p700 tubes (Becton-Dickinson), and for DNA in plain tubes containing K2-EDTA. All tubes were immediately frozen and shipped to Roche for further analysis.

### RT-PCR, protein and copy number determination

RNA from healthy controls was collected in PAXgene tubes and extracted using a MagNA Pure 96 System according to the manufacturer’s instructions (Roche). Patient RNA was extracted from whole blood collected in PaxGene tubes using a Roche in-house manual sample preparation method that uses silica (glass) fleece spin columns. The RNA concentration and quality were determined using NanoDrop. The RNA samples were stored at -80°C before analysis.

The qRT-PCR assay is a single-well reaction utilizing four fluorescent reporter dyes and was designed to run on the cobas z 480 instrument (Roche). One primer set amplifies 199bp each of SMN1 and SMN2 and 145 bp of SMNd7. The 5’primer is positioned in exon 6 and the 3’ primer in exon 8. A second primer set that amplifies 203 bp for the reference gene (RG) is included in each well. The target and reference transcripts are each detected using a sequence-specific probe labeled with a channel-specific reporter dye. Three positive controls and one negative control are included in each run to ensure that the reagents and the assay are functioning properly. The positive controls are: In vitro transcribed RNA of SMN1+RG (SMN1 RNA transcript with an equal copy number of reference gene transcript), SMN2+RG and delta7+RG and a buffer for negative control. Roche Molecular Systems in-house software was used for data analysis. Relative expression (also known as Concentration Ratio, CR) is calculated using 2^-deltaCp, where deltaCp = Target Cp − Reference Cp. The Roche SMN mRNA assay is a fully validated research grade assay which is however, running only on dedicated research machines of the cobas z 480. In order to give the scientific community access to this assay to reproduce the results of this article, we offer to measure mRNA samples sent to the authors at cost until the assay can be made available commercially, within two years after the publication date of this article. Please contact the corresponding author of this publication.

To compare the performance of the Roche cobas assay to the assay previously published in [13]. Briefly, levels of FL and delta7 mRNAs produced by the human *SMN2* transgene were quantified using TaqMan RT-qPCR with 50 ng of total RNA and the *SMN* primers and probes listed in [13]. The *SMN* forward and reverse primers were each used at a final concentration of 0.4 μM. The *SMN* probe was used at a final concentration of 0.15 μM. Human *GAPDH* mRNA was amplified using Applied Biosystems Taqman probe cat No 4310884E. PCR was carried out at the following temperatures for the indicated times: Step 1: 48°C (15 min); Step 2: 95°C (10 min); Step 3: 95°C (15 sec); Step 4: 60°C (1 min); thereafter, Steps 3 and 4 were repeated for 50 cycles.

For protein analysis, whole blood was collected in p700 tubes (Becton-Dickson) and analyzed using an SMN research assay developed by Roche Diagnostics on the Elecsys^®^ platform. Whole blood samples were frozen overnight. Cell lysis was induced by thawing the samples at room temperature. The samples were 1:2 diluted with a Tris-based lysis buffer (Tris-HCl (50 mM), NaCl (300 mM), EDTA (5 mM), Oxy-Pyrion (0.1%), Polidocanol (1%), cOmplete (1 x), pH 7.4) and incubated for 10 min at RT followed by a centrifugation step (5 min at 16,000 x g). The sample supernatants were measured on the Elecsys^®^ cobas^®^ e601 analyzer using the developed SMN research assay. In brief, the samples were incubated with a biotinylated anti- SMN antibody (MAK<SMN1>M-2B1-IgG-Bi(DDS)) and with a ruthenium labelled anti- SMN antibody (MAK<SMN1>M-8-IgG-sulfoBPRu(Osu, Sux)) forming a complex. After addition of streptavidin-coated particles the complex became bound to a solid phase via interaction of biotin and streptavidin. Signal generation is based on the electro-chemiluminescent technology resulting in a signal which is proportionally related to the amount of SMN protein present in the sample. The calibration was based on recombinant human SMN protein (SMN (human), (recombinant) (His-tag); Enzo; Lot: TP321367). A set of five calibrators covering the whole measurement range (0–50,000 pg/ml) was used. The positive controls were based on the same recombinant human SMN protein material (SMN (human), (recombinant) (His-tag); Enzo; Lot: TP321367). Data analysis was done using the OASE software tool version 5.6.2. This Elecsys^®^ assay was compared to an SMN protein assay developed by Pharmoptima (Portage, Michigan, USA) which is commercially available.

SMN copy number was determined by digital PCR using the SMN1 and SMN2 Copy Number Determination Kits (Bio-Rad Laboratories, Hercules, California, USA) according to the manufacturer’s instructions. PCR endpoint analysis for target (SMN1 or SMN2) or reference genes was performed on the QX100TM droplet reader and signal quantitation conducted following the droplet reader and QuantasoftTM v1.6.6 software instruction manual. To confirm technical repeatability, 44 out of the 95 samples from healthy subjects were measured in triplicate with full concordance of the calculated copy number calls. Statistical analysis was done using Matlab 7.12.

## Results

The patients in this study were recruited from two centers in the United States: the University of Utah in Salt Lake City, Utah and the Jasper Clinic in Kalamazoo, Michigan. The demographics of the patients are depicted in Table 1. All 36 patients were distributed over the three types of SMA and ranged in age from under one year up to 61 years old. The copy number of SMN could not be determined for every patient since we were limited by the blood volume allowed to be drawn from children. In two cases the copy number reported by the patients did not match the copy number determined by the digital PCR assay. For these cases we used the digital PCR values. The healthy controls were randomly selected blood donors from the blood bank in Basel, Switzerland.

To reliably analyze the expression levels of the different SMN isoforms, we developed a multiplex RT-PCR assay to differentiate full-length SMN1 from SMN2 and additionally to measure SMNd7 levels in mRNA derived from whole blood. The assay is not designed to provide absolute quantification of mRNA as reported previously ([14], [10]), but to provide a reliable and robust method for monitoring various transcript levels in a sample compared to a reference gene. All mRNA expression data in this study are therefore only relative values compared to a reference gene expressed as concentration ratio (CR) (for calculation see methods section). To have a reproducible and reliable source of mRNA, we used whole blood sampled in PAXgene tubes. These tubes stabilize RNA immediately and minimize variability derived from different storage times after sampling (according to the manufacturer, Becton-Dickinson). The newly developed multiplex qRT-PCR assay has been compared to a previously published qPCR assay ([13]). This assay when applied to human healthy control samples from blood show a high variability. The expression of the reference gene GAPDH shows considerable differences between subjects leading to variability in the corresponding delta CP values for the SMN2 mRNA (S2A Fig and Table S3). The results of the correlation of both assays are shown in S2A Fig. The data show that a group of samples has very different delta Cp values for SMN2. This is driven by the Cp values of GAPDH (Table S3).

**Fig 1:**
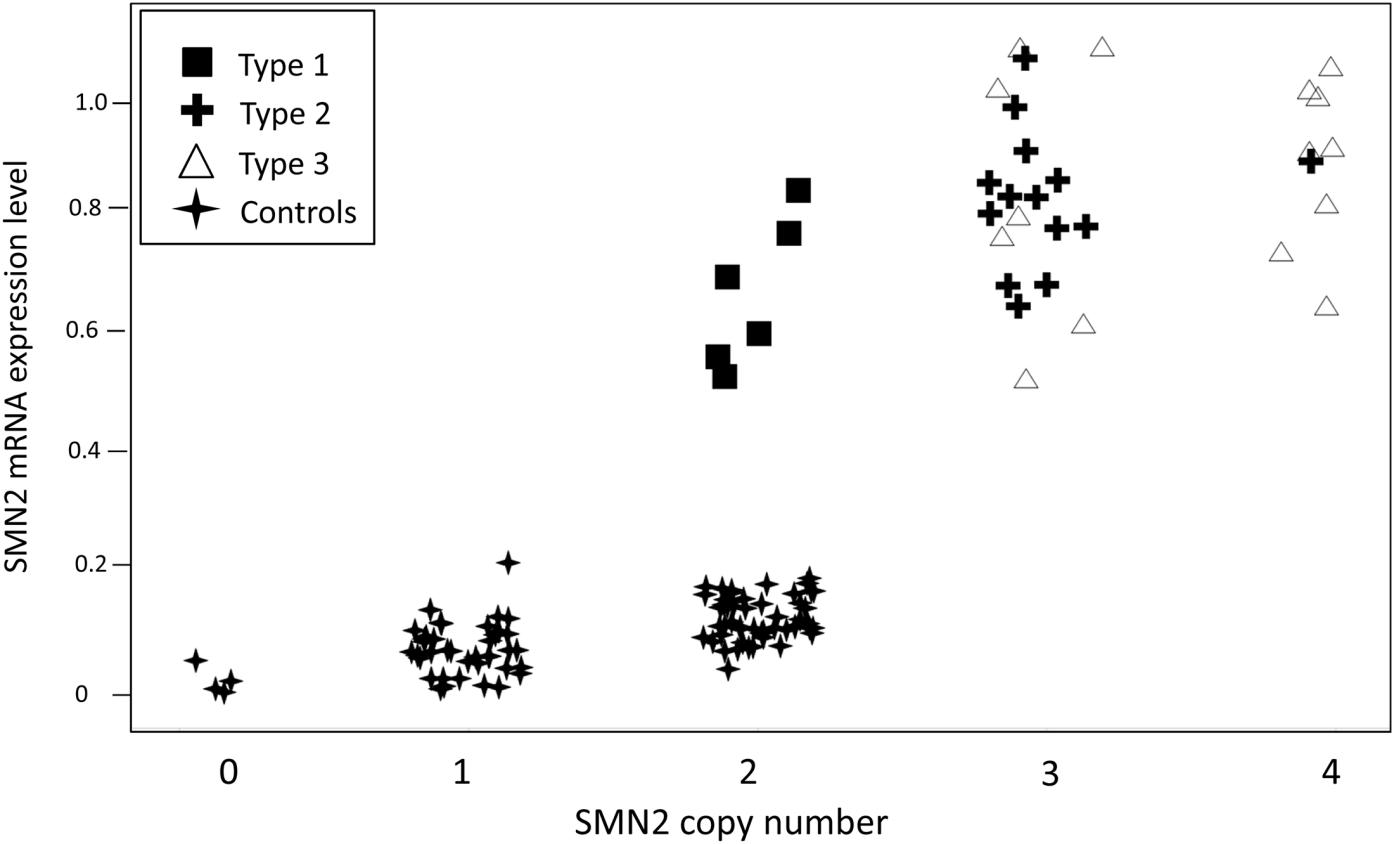
Expression of SMN2 mRNA in SMA patients and healthy controls. SMN2 mRNA was isolated from blood and analyzed using qRT-PCR. Expression level is calculated using 2ˆ-deltaCp of the reference gene. There is a strong overlap of SMN2 mRNA in the different patient groups. Note that in the healthy controls SMN2 levels are lower than levels in patients with the same SMN2 copy number.

We analyzed the copy number of SMN1 and SMN2 using a newly developed digital PCR approach (Bio-Rad Laboratories, Inc.) and correlated the expression level of SMN1 and SMN2 with the copy number of both genes. The distribution of SMN1 and SMN2 copy number in our healthy control group also matches very closely to what has been previously published [15]. We were not able to detect SMN1 mRNA in any of our SMA subjects (S1 Table), Fig 1 shows the mRNA expression levels of full-length SMN2 in our patient cohort in comparison to SMN2 levels in healthy subjects, relative to the reference gene for each copy number of SMN2. SMN2 full-length mRNA expression in blood shows strong overlap among SMA types as described previously ([10]). However, in healthy controls, levels of SMN2 mRNA are substantially lower than in patients even with the same copy number of SMN2. In patients with two copies of SMN2, the expression of SMN2 mRNA is much higher than in controls (Fig 1 shows controls with also two SMN2 copies = 0.128, Type 1 patients with two SMN2 copies = 0.66).

**Fig 2:**
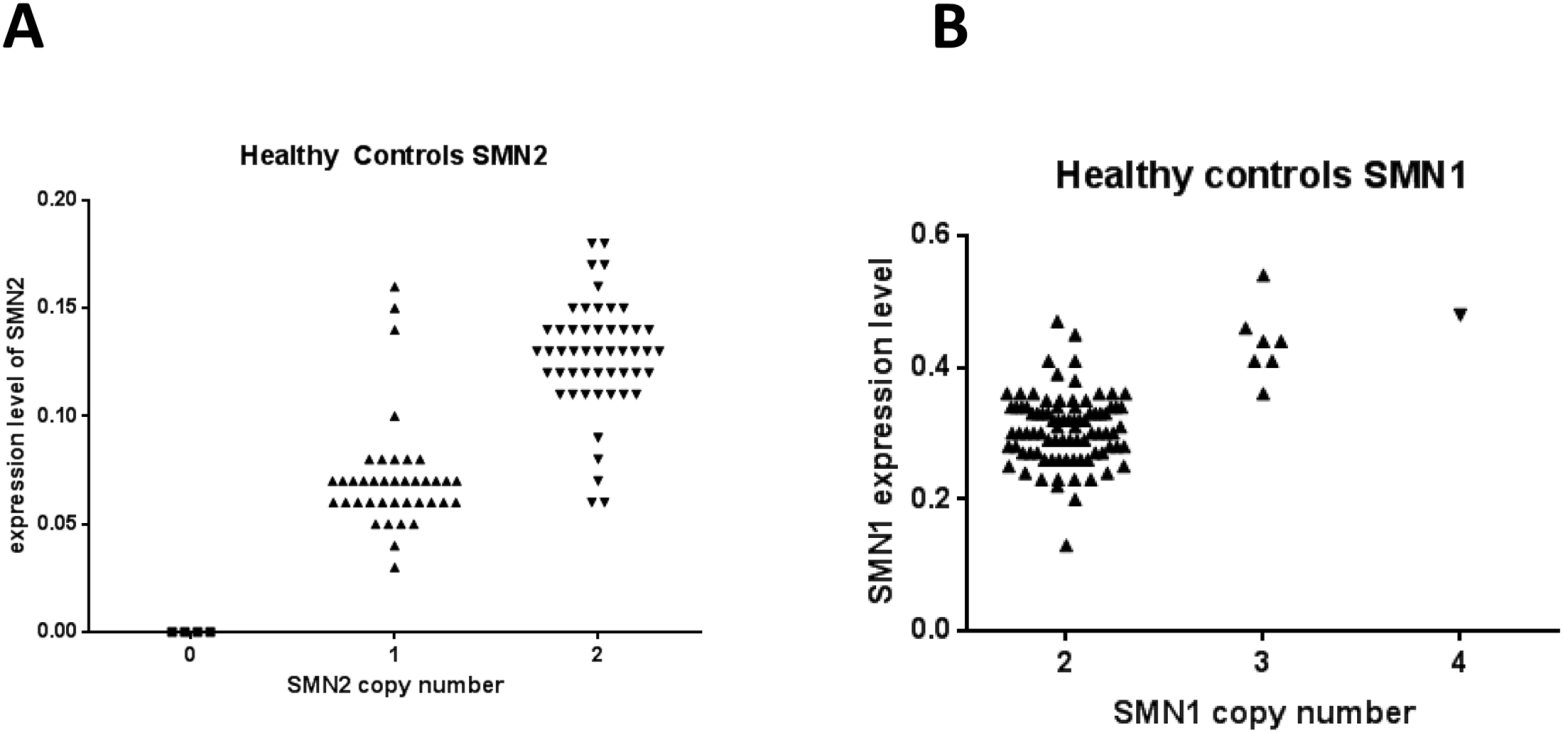
SMN copy number determines expression of SMN in healthy subjects. The copy number and the expression levels of SMN2 are closely linked; two copies show almost double expression of SMN2. Expression level is calculated using 2ˆ-deltaCp of the reference gene. Copy number is determined using digital PCR as described in Methods. Similar for SMN1, increases in copy number lead to increases in mRNA expression.

Interestingly, as shown in Fig 2, there is a very close match of SMN copy number and level of mRNA expression in healthy subjects. Two copies of SMN2 leads to almost doubling of the average mRNA expression levels in the subjects (1 SMN2 copy = 0.069 and 2 SMN2 copies = 0.128) (Fig 2A) and also increasing the copy number of SMN1 leads to a concomitant increase of SMN1 expression (2 copies = 0.31; 3 copies = 0.44; 4 copies = 0.48) (Fig 2B). The association between copy number and transcript level is stronger for SMN2 compared to SMN1. These data suggest that the expression of SMN mRNA in healthy subjects is proportional to the number of gene copies.

In contrast, SMN2 expression levels are much less related to SMN2 copy number in SMA patients (Fig 1). The data show that the average SMN2 mRNA expression values are not very different between copy numbers and that there is a large overlap between groups (2 SMN2 copies = 0.66; 3 SMN2 copies = 0.82; 4 SMN2 copies = 0.90). In particular, the expression levels of SMN2 mRNA between three and four copy numbers are very similar. Interestingly, the data also show that the clinical phenotype is more related to the copy number than to the expression levels of SMN2 in blood. The data suggest that SMN2 expression is differently regulated in patients compared to healthy controls.

In a second step, we looked at SMN protein levels in whole blood of the patients of each SMA Type. For this we have developed a robust assay using whole blood of the SMA patients sampled in p700 tubes. This newly developed assay has been compared with a previously developed assay which is available from PharmOptima (Portage, Michigan, USA). The results of both assays show a very high concordance (S2B Fig). Direct SMN measurement in whole blood samples aimed to avoid potential variability introduced by PBMC isolation and analysis [16] provides an easy and straight forward measurement applicable in large cohorts or clinical trials.

**Fig 3:**
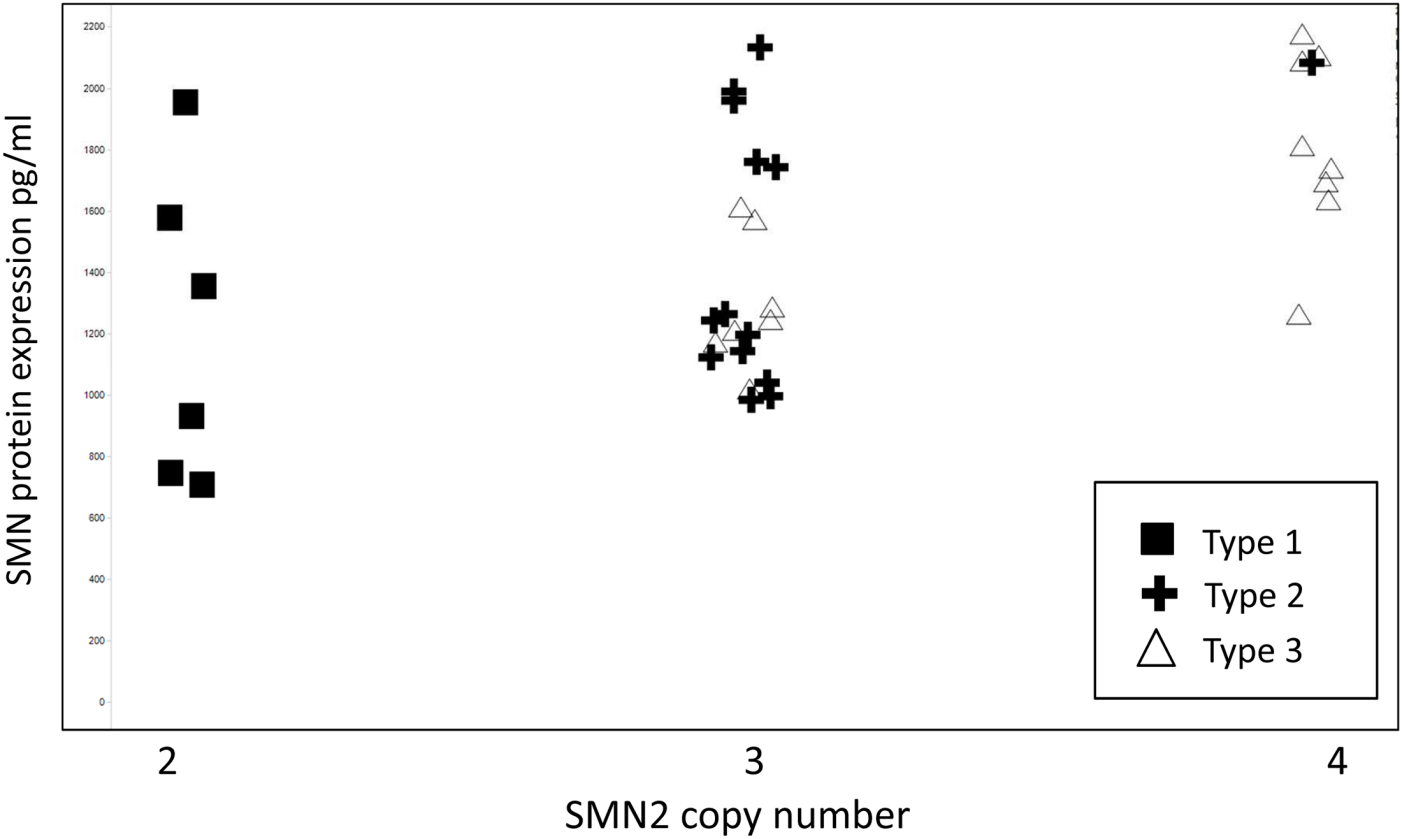
SMN2 copy numbers better predict the clinical phenotype than the expression level. SMN protein was measured in whole blood using the SMN-ECL immunoassay as described. There is a large overlap between protein levels of the different disease Types, but there is a trend for protein increase depending on the copy number. Note that this could be confounded by age.

**Fig 4:**
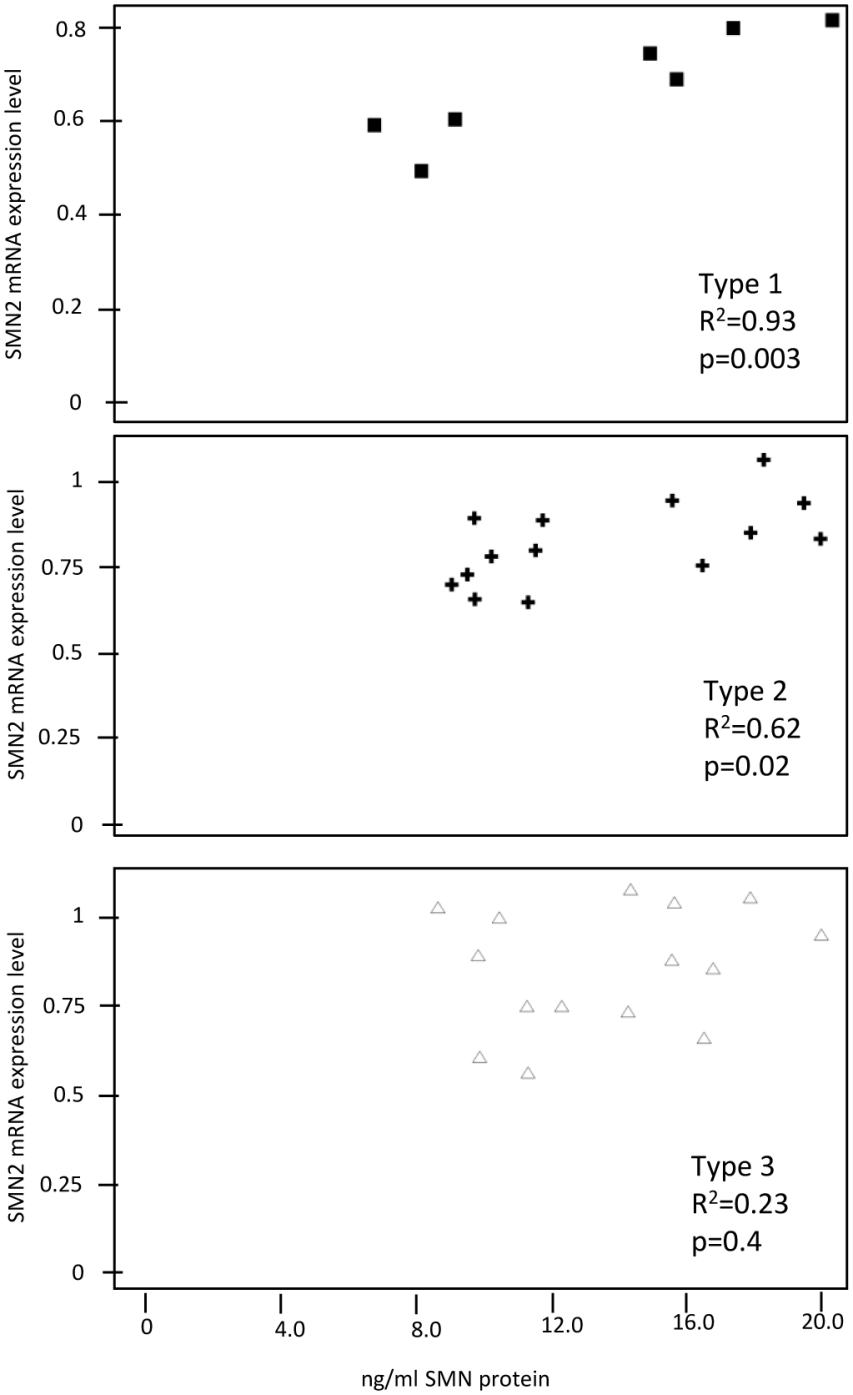
Correlation of protein and mRNA in SMA patients depend on Type. Significant correlation between expression levels of SMN protein and SMN2 mRNA in Type 1 and Type 2 patients but not in Type 3. Statistical analysis was done using Matlab 7.12. Protein levels are in ng/ml (x-axis) and mRNA (y-axis) Expression levels are calculated using 2ˆ-deltaCp of the reference gene.

The data show that SMN protein levels in blood again overlap between different SMA types and the corresponding SMN2 copy numbers better predict the clinical phenotype than the protein expression level (Fig 3). All Type 1 patients have two copies of SMN2 and most of Type 3 patients have four copies. We should note, however, that the expression level could well be influenced by the age of the patients. In particular the three Type 1 patients with the highest expression in Fig 3 are all below one year of age. We did not analyze SMN protein in healthy controls in this study since the protein levels in the healthy population are mostly derived from SMN1 expression. Interestingly, correlation between SMN2 mRNA levels and protein in whole blood is different between the clinical phenotypes (SMA Types). There is a significant correlation in Type 1 patients (R2 = 0.93, p = 0.003) which is not seen in Type 3 patients (Fig 4).

## Discussion

Having reliable assays for analyzing biomarker distribution in the target population is essential for any clinical study targeting increase of SMN protein. In this study we have set up reliable and robust assays for SMN mRNA and protein, which we used to characterize SMA patients of different types and healthy controls. These assays can be applied in clinical practice to study the heterogeneity of SMA and to monitor changes in SMN expression during therapeutic interventions. Both assays have been compared to existing methods for SMN2 mRNA and protein analysis and the results show that both methods are valid to measure SMN mRNA and protein in human blood samples.

The results of the study are in line with previously published data ([10], [14])) showing that the expression of SMN mRNA and protein in blood greatly overlap between different disease types and thus cannot be used as a marker for clinical severity.

Analysis of SMN1 and SMN2 mRNAs and analysis of the corresponding SMN copy number in healthy subjects resulted in new and interesting findings. SMN2 transcript levels separated into two distinct groups corresponding to SMN2 copy number, where doubling of the copy number from one to two almost doubled the mRNA expression levels. We do not know if this tight association between the SMN2 copy number and transcript level would hold with increasing SMN2 copy number as our sample set did not have any healthy individuals with more than two copies of SMN2 gene. In healthy controls, the increased number of SMN1 copies also resulted in increased expression levels of the mRNA. Our data suggest that in healthy controls, SMN1 and SMN2 mRNA expression in blood are not subject to a pronounced transcriptional/mRNA turnover regulation but rather linked to the number of SMN gene copies. However, unlike in healthy individuals, SMN2 mRNA expression in patients is not very closely related to SMN2 copy number. More importantly, SMN2 expression in patients was also higher compared to controls with the same copy number. This argues strongly that expression levels of SMN2 are not only controlled by the number of copies but additional factors are needed to account for it. These findings also suggest that regulation of SMN2 expression in patients is different compared to controls, i.e. SMN2 expression is strongly up-regulated in SMA patients in comparison to healthy individuals. It is important to note that these results are from peripheral blood samples; regulation of SMN1 and SMN2 might be different in motor neurons or muscle tissue.

The data suggest the existence of additional factors regulating SMN expression and influencing the clinical phenotype of the disease. It has been shown previously that SMN2 copy number only has a modest relation to clinical severity and to expression level ([10]). SMA patients with the same copy number of SMN2 and even closely related siblings can have different severities and clinical phenotype of the disease ([17]). These data have been well reproduced in our study, in particular for patients with Type 2 and Type 3 SMA. This phenotypic heterogeneity and the selective vulnerability of certain types of motor neurons in SMA could potentially be explained by a regulatory mechanism for SMN expression that varies between cell types and tissues. SMN expression in affected tissues, such as spinal cord and skeletal muscle, could be regulated differently compared to SMN expression in peripheral blood. Further studies are needed to address this question, but here we provide the tools to study this phenomenon in more detail.

In this study, we used a protocol for analyzing the protein content in all cells present in 3 mL of whole blood samples. Consistent with previously published work (Crawford et al, 2012), our data show an overlap in SMN protein levels for patients with varying degrees of clinical severity. We found a correlation between SMN2 mRNA levels and protein in Type 1 and Type 2 patients but not in Type 3 patients.

The lack of correlation in Type 3 patients could be due to the average age of the patient group; young Type 1 patients are shown to have the highest protein levels. Further studies, with a greater number of patients of various ages and also longitudinal studies are necessary to address these questions reliably. It may be necessary to examine different tissues, such as affected motor neurons or muscle, to more clearly understand the correlation of SMN mRNA with protein and to relate it to the clinical phenotype.

In summary, we have characterized the expression of peripheral SMN in a group of 36 SMA patients with varying disease severities and 95 healthy adult controls. The healthy subjects in this study are neither age- nor gender-matched which clearly limits the conclusions in particular for very young patients. There could be an age dependent regulation of SMN expression which cannot reliably be addressed in this limited data set. The data show a clear up-regulation of SMN2 mRNA expression in blood of SMA patients and also shows that expression of SMN is very differently regulated in patients and healthy subjects.

These results might be used in subsequent studies to analyze genetic, epigenetic or environmental factors influencing the expression of SMN and provide potential new targets for therapeutic intervention. Moreover, having robust and reliable assays to measure SMN protein and mRNA assays in place provide the tools to further study the regulation of SMN expression in patients and to monitor the activity of disease modifying treatments targeting in particular the increase of SMN expression.

## Supporting Information

S1 Fig Transparent reporting of clinical trials CONSORT 2010 Flow Diagram. (DOCX)

S2 Fig Comparison of newly developed SMN mRNA and SMN protein assays with previously published assays. Fig A. Comparison of Roche qRT-PCR assay on COBAS (SMN2 mRNA assay B) with SMN2 mRNA assay B ([13]et al). Note the group of values at the left side of the graph which are moved due to low expression of the GAPDH reference gene as used by Naryshkin et al and a resulting decrease in the delta Cp values. DeltaCp is calculated by subtracting the Cp value of SMN2 by Cp of the respective reference gene. Fig B. Comparison of SMN assay developed on the Roche Elecsys^®^ platform with Pharmoptima, (Portage, Michigan) assay. Both assays show good concordance, different absolute levels of protein may be the result of different protein standards.
(TIF)

S1 Protocol Study protocol. (PDF)

S1 Table Raw data of the assay. All data and values from the different assays of SMA patients and Healthy controls (HC). Abbreviations are: SMN1FL = SMN1 full length, SMN2FL = SMN2 full length SMN2d7 = SMN delta Exon 7, SMN-RG = Reference Gene used for the SMN Cobas assay. CR = Relative expression (also known as Concentration Ratio, CR) is calculated using 2^-deltaCp, where deltaCp = Target Cp − Reference Cp.
(PDF)

S1 TREND Checklist TREND Checklist. (DOCX)
