# Supplementary material for: Biomarker for Spinal Muscular Atrophy: Expression of SMN in Peripheral Blood of SMA Patients and Healthy Controls
Source: PLoS One. 2015 Oct 15;10(10):e0139950. doi: 10.1371/journal.pone.0139950 (PMC4607439; doi:10.1371/journal.pone.0139950)
Supplement: S1 Fig — (DOCX) [file pone.0139950.s001.docx]

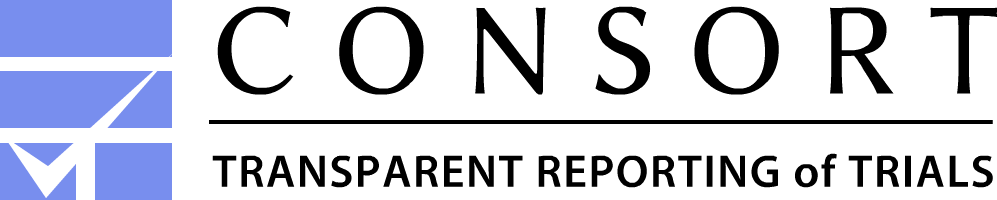


**CONSORT 2010 Flow Diagram for:**

**A MULTI CENTER STUDY TO COLLECT SAMPLES FROM SMA PATIENTS FOR BIOMARKER ANALYSIS**

Assessed for eligibility (n= 36)

Allocated to biomarker sampling (n=36)

- RNA samples (n=36)
- Protein samples (n=36)
- DNA samples (n=26)

Not from all patients sufficient blood could be drawn for all sampling

Included (n=36)

For single visit

Excluded (n=0)

♦  Not meeting inclusion criteria (n=0)

♦  Declined to participate (n=0)

♦  Other reasons (n=0)

Analysed (n=36 )
♦ Excluded from analysis (give reasons) (n=0)
