# Supplementary figures and images for: Biomarker for Spinal Muscular Atrophy: Expression of SMN in Peripheral Blood of SMA Patients and Healthy Controls
Source: PLoS One. 2015 Oct 15;10(10):e0139950. doi: 10.1371/journal.pone.0139950 (PMC4607439; doi:10.1371/journal.pone.0139950)

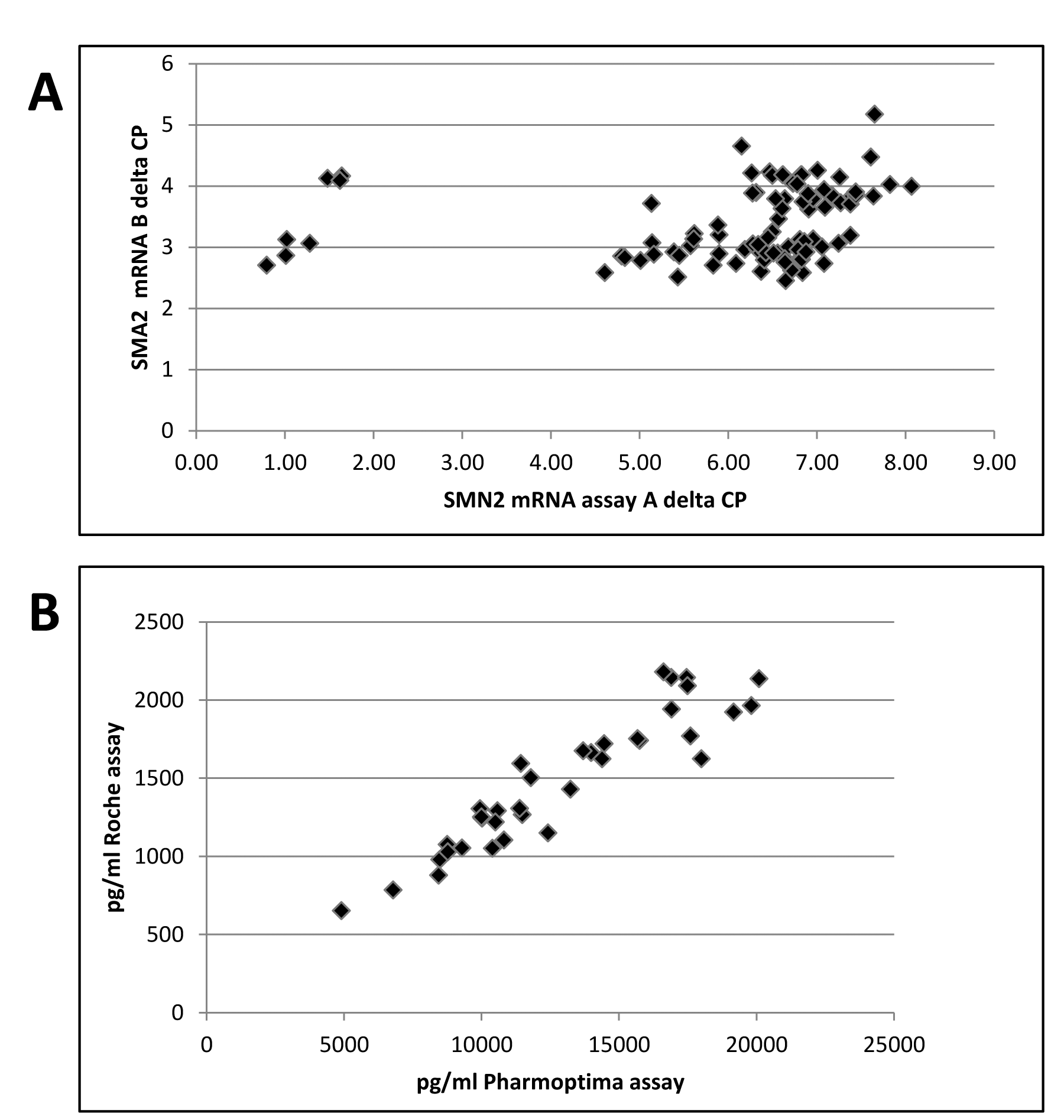

Supplement: S2 Fig — Fig A. Comparison of Roche qRT-PCR assay on COBAS (SMN2 mRNA assay B) with SMN2 mRNA assay B ([13]et al). Note the group of values at the left side of the graph which are moved due to low expression of the GAPDH reference gene as used by Naryshkin et al and a resulting decrease in the delta Cp values. DeltaCp is calculated by subtracting the Cp value of SMN2 by Cp of the respective reference gene. Fig B. Comparison of SMN assay developed on the Roche Elecsys® platform with Pharmoptima, (Portage, Michigan) assay. Both assays show good concordance, different absolute levels of protein may be the result of different protein standards. (TIF) [file pone.0139950.s002.tif]
