# Supplementary material for: Biomarker for Spinal Muscular Atrophy: Expression of SMN in Peripheral Blood of SMA Patients and Healthy Controls
Source: PLoS One. 2015 Oct 15;10(10):e0139950. doi: 10.1371/journal.pone.0139950 (PMC4607439; doi:10.1371/journal.pone.0139950)
Supplement: S1 Protocol — (PDF) [file pone.0139950.s003.pdf]

PROTOCOL

**TITLE:**	**A MULTI CENTER STUDY TO COLLECT SAMPLES FROM SMA PATIENTS FOR BIOMARKER ANALYSIS**
**PROTOCOL NUMBER:**	BE29002
**VERSION NUMBER:**	1
**EUDRACT NUMBER:**	n/a
**IND NUMBER:**	n/a
**TEST PRODUCT:**	n/a
**MEDICAL MONITOR:**	XXXXXXX
**SPONSOR:**	F. Hoffmann-La Roche Ltd
**DATE FINAL:**	See electronic date stamp below

FINAL PROTOCOL APPROVAL

CONFIDENTIAL STATEMENT

The information contained in this document, especially any unpublished data, is the property of F.Hoffmann-La Roche Ltd (or under its control) and therefore is provided to you in confidence as an investigator, potential investigator, or consultant, for review by you, your staff, and an applicable Ethics Committee or Institutional Review Board. It is understood that this information will not be disclosed to others without written authorization from Roche except to the extent necessary to obtain informed consent from study participants.

TABLE OF CONTENTS

1. BACKGROUND AND RATIONALE ... 7

2. OBJECTIVES OF THE STUDY ... 8

2.1 Primary Objective ... 8

2.2 Secondary Objectives ... 8

3. STUDY DESIGN ... 8

3.1 Overview of Study Design ... 8

3.1.1 End of Study Definition ... 8

4. STUDY POPULATION ... 8

4.1 Target Population ... 8

4.1.1 Recruitment Procedures ... 9

4.2 Inclusion Criteria ... 9

4.3 Exclusion Criteria ... 9

4.4 Criteria for Premature Withdrawal ... 9

4.5 Concomitant Medication and Treatment/Special Dietary Requirements ... 10

5. SCHEDULE OF ASSESSMENTS AND PROCEDURES ... 10

5.1 Screening and Enrollment ... 11

5.1.1 Screening Examination and Eligibility Screening Form 11

5.1.2 Enrollment procedure ... 11

5.2 Study Procedures/Assessments ... 11

5.2.1 Study Procedures ... 11

5.2.1.1 Biomarker Assessments ... 13

6. SAFETY INSTRUCTIONS AND GUIDANCE ... 13

6.1 Adverse Events ... 14

6.1.1 Adverse Events ... 14

6.1.2 Intensity ... 15

6.1.3 Serious Adverse Events (Immediately Reportable to Roche) 15

6.2 Methods and Timing for Capturing and Assessing Safety Parameters ... 15

6.2.1 Eliciting Adverse Event Information ... 16

6.2.2 Assessment of Severity of Adverse Events 16
6.2.3 Assessment of Causality of Adverse Events.......................... 16
6.2.4 Procedures for Recording Adverse Events 16
6.2.4.1 Deaths 17
6.3 Immediate Reporting Requirements from Investigator to Sponsor 17
6.3.1 Emergency Medical Contacts 18
6.4 Follow-Up of Patients after Adverse Events........................... 18
6.4.1 Investigator Follow-Up 18
7. STATISTICAL CONSIDERATIONS AND ANALYTICAL PLAN................... 18
7.1 Safety Data Analysis 19
7.1.1 Adverse Events.. 19
7.2 Replacement Policy (Ensuring Adequate Numbers of Evaluable Patients) 19
7.3 Assignment of Preferred Terms and Original Terminology 19
8. ETHICAL ASPECTS 20
8.1 Local Regulations/Declaration of Helsinki 20
8.2 Informed Consent 20
8.3 Independent Ethics Committees/Institutional Review Board 20
9. CONDITIONS FOR MODIFYING THE PROTOCOL.................................. 21
10. CONDITIONS FOR TERMINATING THE STUDY 21
11. STUDY DOCUMENTATION, CRF OR ECRF AND RECORD KEEPING 21
11.1 Investigator’s Files/Retention of Documents.......................... 21
11.2 Source Documents and Background Data 22
11.3 Audits and Inspections... 22
11.4 Electronic Case report Forms 22
12. MONITORING THE STUDY.. 22
13. CONFIDENTIALITY OF TRIAL DOCUMENTS AND PATIENT RECORDS 23

LIST OF TABLES

Table 1 Number of Samples and Approximate Amount of Blood to be collected from Each Patient.. 13
Table 2 Adverse Event Severity Grading Scale 16

GLOSSARY OF ABBREVIATIONS

AE	Adverse event
ANOVA	Analysis of variance
ASR	Annual safety report
CA	Competent authority
CFR	Code of Federal Regulations
CI	Confidence interval
CRO	Contract research organization
eCRF	Electronic case report form
EDC	Electronic data capture
Eform	Electronic form
ESF	Eligibility screening form
FDA	Food and Drug Administration
GCP	Good Clinical Practice
ICH	International Conference on Harmonization
IEC	Independent Ethics Committee
IRB	Institutional Review Board
LPLV / LSLV	Last patient last visit / last subject last visit
MedDRA	Medical Dictionary for Regulatory Activities Terminology
mRNA	messenger Ribonucleic Acid
MOS	Margin of safety
SAE	Serious adverse event.
SI	Système international d’unités (International system of units)

GLOSSARY OF ABBREVIATIONS

SoATs	Schedule of Assessment Tables
SMT	Study management team
SUSAR	Suspected Unexpected Serious Adverse Reaction
TBD	To be determined
USAN	United States Adopted Name
HFMS	Hammersmith Functional Motor Scale
SMA	Spinal Muscular Atrophy
SMN	Survival motor neuron (gene or protein)

PART I: STUDY DESIGN AND CONDUCT

1. BACKGROUND AND RATIONALE

Spinal Muscular Atrophy (SMA) is a common inherited pediatric neuromuscular disorder. There currently is no cure and there are no therapeutics approved to slow progression of the disease. SMA is characterized by a loss of alpha motor neurons in the spinal cord, severe atrophy of proximal muscles and progressive debility and disability due to respiratory, gastrointestinal and functional complications of the disease. The disease is caused by a lack of the survival motor neuron (SMN) protein, which is coded by the SMN1 gene. Most SMA patients have a deletion of the SMN1 gene, but a small minority of patients have smaller point mutations in the SMN1 gene. Humans have a copy of a nearly identical gene, SMN2, which can compensate to some degree for the loss of SMN1 in SMA patients.

While several new therapies for SMA are poised to enter the clinic, there are a number of challenges to executing SMA trials that must be addressed. The putative target tissue for SMA treatment is the spinal cord, a tissue that cannot be sampled directly to evaluate drug effects. Thus it is important to develop meaningful measurements from accessible tissues like blood that relate to changes in the spinal cord. Also, SMA is a disease that presents in a heterogeneous manner with a spectrum of motor function (from not being able to sit up to not being able to walk) and variability in disease onset (from birth to the third decade of life). The variability of SMA phenotypes requires careful consideration about which patients are best to enroll in each specific drug trial. Finally, although SMA is a relatively common orphan disease, recruitment of patients for the number of candidate therapies is expected to become rate-limiting for the development of therapeutics.

Several of the challenges described above can be met by developing ‘biomarkers’ that can help predict responses to drugs in SMA patients in simple blood specimens. A biomarker is a characteristic that is objectively measured and evaluated, and is an indicator of a biologic process. The potential advantages for developing biomarkers for SMA are many and largely can be applied to any new drugs that are tested, and the uses are listed below:

- May allow replacement of a distal endpoint with a proximal endpoint, potentially shortening the development time of new therapeutic modalities
- Measures in blood may be done more frequently and easily than costly and complex functional tests
- May have greater precision
- May increase the measured dynamic range of a disease process or treatment effect compared to clinical metrics
- May provide a rationale for the selection of a patient population more likely to respond positively to drugs
- May result in reduced sample size requirements for clinical studies
- May lead to expedited decisions concerning the validity of therapeutic interventions

Thus, the development of SMA biomarkers will have important implications in therapeutics development timelines and more efficiently allocate patient resources into studies with the greatest probability of success. **OBJECTIVES OF THE STUDY**

2.1 PRIMARY OBJECTIVE

- To refine and validate mRNA assays for SMN1 and SMN2 detection in blood from SMA patients.
- To measure SMN protein in whole blood and in isolated lymphocytes in SMA patients.
- To establish correlations between mRNA and protein levels in blood and SMN2 copy number in SMA patients
- To validate protocols for sample collection, SMN mRNA and protein quantitation in SMA patients

2.2 SECONDARY OBJECTIVES

To collect blood samples that could be used to continue validation of new mRNA or protein biomarkers for SMA. Any new SMA biomarker could ultimately be used as a tool in clinical trials to determine if patient functional status is changing in response to drug treatment.

3. STUDY DESIGN

3.1 OVERVIEW OF STUDY DESIGN

This study will be a single visit study enrolling up to 40 patients. The study will enroll patients that are self-identified SMA patients.

This is a multi center study to be conducted in the USA. An additional site(s) may be included for back-up purposes and may be activated if needed. *Administrative and Contact Information and List of Investigators* are provided separately.

3.1.1 End of Study Definition

The end of the trial is defined as the date of the last visit of the last patient undergoing the trial.

4. STUDY POPULATION

4.1 TARGET POPULATION

Three groups of SMA patients may be enrolled according to the classifications below:

- Patients with type I SMA (max of 10)
- Patients with type II SMA (minimum of 5 and maximum of 15)
- Patients with type III SMA (minimum of 5 and maximum of 15)

4.1.1 Recruitment Procedures

The Principal Investigator (or designee) and the study coordinator will have responsibility for patient recruitment in conjunction with the local chapter of the Families of SMA who will pre-identify patients within their network.

4.2 INCLUSION CRITERIA

A patient may be included if the answer to all of the following statements is ‘yes’:

1. Self-identified as 5q-autosomal recessive SMA type I, II, or III as judged by their neurologist upon diagnosis.

2. Ability and willingness to provide blood samples.

3. Willingness (by the patient or patient’s parents or legal guardian) to complete to their best ability a questionnaire which requests specific clinical and genetic information.

4. Able to participate and willing to give written informed consent or assent. Informed consent will be obtained from the patient, or the patient’s parent or legal guardian.

4.3 EXCLUSION CRITERIA

A patient will be excluded if the answer to any of the following statements is ‘yes’:

1. Any known genetic condition other than Spinal Muscular Atrophy, unless it is not interfering with the purpose of this study based on the Sponsor’s judgment.
2. Participation in a clinical trial (except observational studies) within the previous 14 days.
3. Donation of blood or significant blood loss within three months prior to screening.
4. Concomitant disease or condition that could interfere with, or treatment of which might interfere with, the conduct of the study, or that would, in the opinion of the investigator, pose an unacceptable risk to the patient in this study.

4.4 CRITERIA FOR PREMATURE WITHDRAWAL

Patients have the right to withdraw from the study at any time for any reason. In the event that a patient decides to prematurely discontinue from the study, he/she should be asked if he/she can still be contacted for further information. The outcome of that discussion should be documented in both the medical records and in the eCRF.

When applicable, patients should be informed of circumstances under which their participation may be terminated by the investigator without the patient's consent. The investigator may withdraw patients from the study in the event of intercurrent illness, adverse events, lack of compliance with the study and/or study procedures (e.g., study visits) or any other reason where the investigator feels it is in the best interest of the

patient to be terminated from the study. Reasons for withdrawal must be documented and explained to the patient. It is understood by all concerned that an excessive rate of withdrawals can render the study uninterpretable; therefore, unnecessary withdrawal of patients should be avoided. Should a patient decide to withdraw, all efforts should be made to complete and report the observations as thoroughly as possible.

If the reason for removal of a patient from the study is an AE related to study procedures, the principal specific event must be recorded on the eCRF. The patient should be followed until the AE is resolved, if possible.

4.5 CONCOMITANT MEDICATION AND TREATMENT/SPECIAL DIETARY REQUIREMENTS

All medications (prescription and over-the-counter [OTC]) taken within 30 days before the screening / study visit will be recorded on the appropriate eCRF.

5. SCHEDULE OF ASSESSMENTS AND PROCEDURES

At the time of enrollment, the research staff will use the patient questionnaire to obtain self-declared clinical history and other information:

- Gender
- Age
- Race
- SMA Type (I or II or III)
- Age at onset
- SMN2 copy number (if known by the patient)
- Hammersmith Functional Motor Scale (HFMS) or equivalent (if known)
- If the patient has taken any potentially SMN-enhancing drugs in the past 7 days, eg. valproic acid, phenylbutyrate, hydroxyurea, carnitine, riluzole, creatine, oral albuterol
- Patient’s current level of function (sitting without support, rolling, crawling, standing, walking)
- Highest motor function achieved (sitting without support, rolling, crawling, standing, walking)

5.1 SCREENING AND ENROLLMENT

5.1.1 Screening Examination and Eligibility Screening Form

All patients or their parent/legal guardian must sign and date the most current IRB/IEC-approved written informed consent before any study specific assessments or procedures are performed. Children older than seven years will be asked to sign an assent. An original signed consent form will be retained by the investigator and the patient will receive a copy to take home.

An Eligibility Screening Form (ESF) documenting the investigator's assessment of each screened patient with regard to the protocol's inclusion and exclusion criteria is to be completed and signed by the investigator (or designee).

A screen failure log must be maintained by the investigator.

Ethnicity of patients will be recorded since this information might be important to evaluate a potential impact of ethnic factors on biomarkers (see also ICH Guideline E5(R1)).

5.1.2 Enrollment procedure

Patients cannot commence enrollment procedures until all entry criteria have been fulfilled.

Under no circumstances will patients who enroll in this study and have provided a blood sample as specified, be permitted to re-enroll in the study. A patient Enrollment and Identification Code List must be maintained by the investigator.

5.2 STUDY PROCEDURES/ASSESSMENTS

5.2.1 Study Procedures

Signed and dated IRB-approved informed consent or assent must be obtained from the patient, or the patient's parent or guardian, before any study-specific screening procedures are performed. The possible benefits and complications of participation will be explained in detail. Every effort will be made to ensure that the patient and parent/guardian fully comprehend the nature of the study and the details of his or her participation. A copy of the consent form will be provided to the parent/guardian and patient. The informed consent process must be documented in the patient's study record.

After the inclusion and exclusion criteria have been assessed and a patient was judged to be eligible for the study, sample collection for the study will take place on the same day and during the same visit.

Blood Sample Collection

The following mandatory samples will be collected in order of priority:

- Blood for RNA Expression Profiling
  Blood (approximately 2.5 mL collected in PAXgene vacutainers) for RNA isolation will be obtained from every patient.

- Whole blood for protein analysis
  One blood (approximately 3 mL) sample for SMN protein analysis will be collected from every patient. Collected samples may be used for exploratory studies in order to correlate SMN protein with other biomarkers of SMA.

- Whole Blood for DNA Analysis
  A whole Blood sample of approximately 3 mL will be taken from every patient for DNA extraction and analysis of genetic information. The genetic information collected will be related to SMN1 and SMN2 and may include additional genes in the disease pathway of SMA.

- Plasma for biomarker analysis
  Blood (one approximately 6.0 mL sample in EDTA) for plasma isolation will be obtained. These samples will be used for biomarker assays which may include IGF and IGF binding proteins, the SMA Multi-analyte panel or other candidate SMA biomarkers.

- Serum for biomarker analysis
  Blood (one approximately 6.0 mL sample in a plain tube without EDTA) for serum isolation will be obtained. These samples will be used for biomarker assays which may include IGF and IGF binding proteins, the SMA Multi-analyte panel or other candidate SMA biomarkers.

For sampling procedures, storage conditions and shipment instructions see study Sample Handling and Logistics Manual.

Total Blood Loss

A total of up to 21 mL of blood will be drawn from each patient. In case patients are considered too fragile or below 15kg of body weight, only samples for RNA Expression profiling and protein analysis will be collected.

After collection of the blood samples and completion of the questionnaires, patients will get dismissed and their participation in the study is considered complete. No specific follow-up assessment will take place.

Table 1 Number of Samples and Approximate Amount of Blood to be collected from Each Patient

Test	Day 1	Total No. of Samples	Approx. No. of mL per Sample	Sub-Total mL of Blood
Blood for RNA expression profiling	1	1	2.5	2.5
Whole blood for protein analysis	1	1	3	3
Whole blood for DNA analysis	1	1	3	3
Plasma for biomarker analysis	1	1	6	6
Serum for biomarker analysis	1	1	6	6
Approximate Total Blood Volume				20.5

5.2.1.1 Biomarker Assessments

All biomarkers which will be explored in this study are related to SMA. Measurements of biomarkers will comprise mRNAs of SMN1 and SMN2 and SMN protein. Measurements include also additional mRNAs or proteins to be used as standards for quantification. Biomarker assessment may also include candidate biomarkers for SMA related to the clinical phenotype.

Genotyping will be performed on each patient including but not limited to determining the copy number of SMN2 since the severity of SMA is modulated by the number of copies of SMN2 which in turn modulates the levels of SMN protein. The genetic information collected will be related to SMN1 and SMN2 and may include additional genes in the disease pathway of SMA.

6. SAFETY INSTRUCTIONS AND GUIDANCE

Safety assessments will consist of monitoring and recording adverse events, including serious adverse events and non-serious adverse events of special interest.

Certain types of events require immediate reporting to the Sponsor, as outlined in Section 6.3.

6.1 ADVERSE EVENTS

6.1.1 Adverse Events

According to the ICH guideline, as patients will not receive an investigational drug in the study, only study procedure-related adverse events and events of interest will be reported.

Study procedure-related adverse events are events which the investigator regards to be clinically relevant and related directly to the procedures of the study e.g. blood sampling.

Events of interest include events leading to death, hospitalizations, any procedure complications or disease progression that appears more rapid than expected.

All AEs encountered during the study and for up to 28 days after the study visit which are clinically relevant and related directly to the procedures within this protocol will be reported on the AE form of the eCRF. At the study completion/early termination visit, the investigator should instruct each patient to report to the investigator any subsequent adverse events that the patient's personal physician believes could be related to study procedures.

The investigator should notify the Sponsor of any death, serious adverse event, or other adverse event of concern occurring at any time after a patient has discontinued study participation if the event is believed to be related to study procedures.

For Good Clinical Practice, an adverse event is any untoward medical occurrence in a clinical investigation subject administered a pharmaceutical product, regardless of causal attribution. An adverse event can therefore be any of the following:

- Any unfavorable and unintended sign (including an abnormal laboratory finding), symptom, or disease temporally associated with the use of a medicinal product, whether or not considered related to the medicinal product.
- Any new disease or exacerbation of an existing disease (a worsening in the character, frequency, or severity of a known condition). Recurrence of an intermittent medical condition (e.g., headache) not present at baseline.
- Any deterioration in a laboratory value or other clinical test (e.g., ECG, X-ray) that is associated with symptoms or leads to a change in study treatment or concomitant treatment or discontinuation from study drug.
- Adverse events that are related to a protocol-mandated intervention, including those that occur prior to assignment of study treatment (e.g., invasive screening procedures such as biopsies).

6.1.2 Intensity

Intensity of AEs will be graded on a three-point scale (mild, moderate, severe) and reported in detail on the eCRF. Table 2 provides guidance for assessing adverse event severity.

6.1.3 Serious Adverse Events (Immediately Reportable to Roche)

A serious adverse event is any adverse event that meets any of the following criteria:

- Fatal (i.e., the adverse event actually causes or leads to death)
- Life threatening (i.e., the adverse event, in the view of the investigator, places the patient at immediate risk of death)

    This does not include any adverse event that had it occurred in a more severe form or was allowed to continue might have caused death.

- Requires or prolongs inpatient hospitalization
- Results in persistent or significant disability/incapacity (i.e., the adverse event results in substantial disruption of the patient’s ability to conduct normal life functions)
- Significant medical event in the investigator's judgment (e.g., may jeopardize the patient or may require medical/surgical intervention to prevent one of the outcomes listed above)

The terms “severe” and “serious” are not synonymous. Severity refers to the intensity of an adverse event (rated as mild, moderate, or severe); the event itself may be of relatively minor medical significance (such as severe headache without any further findings).

Severity and seriousness need to be independently assessed for each adverse event recorded on the eCRF.

Serious adverse events are required to be reported by the investigator to the Sponsor immediately (i.e., no more than 24 hours after learning of the event; see Section 6.3 for reporting instructions).

6.2 METHODS AND TIMING FOR CAPTURING AND ASSESSING SAFETY PARAMETERS

The investigator is responsible for ensuring that all procedure-related adverse events are recorded on the Adverse Event eCRF and reported to the Sponsor in accordance with instructions provided**.** For each adverse event recorded on the Adverse Event eCRF, the investigator will make an assessment of seriousness.

6.2.1 Eliciting Adverse Event Information

A consistent method of non-directive questioning should be adopted for eliciting adverse event information at all patient evaluation timepoints. Examples of non-directive questions include the following:

"How have you felt since your last clinic visit?"

"Have you had any new or changed health problems since you were last here?"

6.2.2 Assessment of Severity of Adverse Events

Table 2 provides guidance for assessing adverse event severity.

Table 2 Adverse Event Severity Grading Scale

Severity	Description
Mild	Discomfort noticed, but no disruption of normal daily activity
Moderate	Discomfort sufficient to reduce or affect normal daily activity
Severe	Incapacitating with inability to work or to perform normal daily activity

Note: Regardless of severity, some events may also meet seriousness criteria. Refer to definition of a serious adverse event (see Section 6.1.3).

6.2.3 Assessment of Causality of Adverse Events

Investigators should use their knowledge of the patient, the circumstances surrounding the event, and an evaluation of any potential alternative causes to determine whether or not an adverse event is considered to be related to the procedure, indicating "yes" or "no" accordingly. The following guidance should be taken into consideration:

- Temporal relationship of event onset to the study procedure
- Known association of the event with the study procedures
- Known association of the event with the disease under study
- Presence of risk factors in the patient or use of concomitant medications known to increase the occurrence of the event
- Presence of non-treatment-related factors that are known to be associated with the occurrence of the event

6.2.4 Procedures for Recording Adverse Events

Investigators should use correct medical terminology/concepts when recording adverse events on the Adverse Event eCRF. Avoid colloquialisms and abbreviations.

Only one adverse event term should be recorded in the event field on the Adverse Event eCRF.

6.2.4.1 Deaths

All deaths that occur during the protocol-specified adverse event reporting period (see Section 6.1.1), regardless of relationship to study procedures, must be recorded on the Adverse Event eCRF and immediately reported to the Sponsor (see Section 6.1.1).

Death should be considered an outcome and not a distinct event. The event or condition that caused or contributed to the fatal outcome should be recorded as the single medical concept on the Adverse Event eCRF. Generally, only one such event should be reported. The term **"sudden death"** should only be used for the occurrence of an abrupt and unexpected death due to presumed cardiac causes in a patient with or without preexisting heart disease, within 1 hour of the onset of acute symptoms or, in the case of an unwitnessed death, within 24 hours after the patient was last seen alive and stable. If the cause of death is unknown and cannot be ascertained at the time of reporting, **"unexplained death"** should be recorded on the Adverse Event eCRF. If the cause of death later becomes available (e.g., after autopsy), "unexplained death" should be replaced by the established cause of death.

6.3 IMMEDIATE REPORTING REQUIREMENTS FROM INVESTIGATOR TO SPONSOR

Certain events require immediate reporting to allow the Sponsor to take appropriate measures to address potential new risks in a clinical trial. The investigator must report such events to the Sponsor immediately; under no circumstances should reporting take place more than 24 hours after the investigator learns of the event. The following is a list of events that the investigator must report to the Sponsor within 24 hours after learning of the event, regardless of relationship to study procedure:

- Serious adverse events
- Non-serious adverse events of special interest
- Pregnancies

The investigator must report new significant follow-up information for these events to the Sponsor immediately (i.e., no more than 24 hours after becoming aware of the information). New significant information includes the following:

- New signs or symptoms or a change in the diagnosis
- Significant new diagnostic test results
- Change in causality based on new information
- Change in the event's outcome, including recovery
- Additional narrative information on the clinical course of the event

Investigators must also comply with local requirements for reporting serious adverse events to the local health authority and IRB/EC.

6.3.1 Emergency Medical Contacts

MEDICAL MONITOR (ROCHE MEDICAL RESPONSIBLE) CONTACT INFORMATION

Primary Contact

Medical Monitor: xxxxxxxxxxx

Telephone No.: [redacted]

Mobile Telephone No.: [redacted]

To ensure the safety of study patients, an Emergency Medical Call Center Help Desk will access the Roche Medical Emergency List, escalate emergency medical calls, provide medical translation service (if necessary), connect the investigator with a Roche Medical Monitor, and track all calls. The Emergency Medical Call Center Help Desk will be available 24 hours per day, 7 days per week. Toll-free numbers for the Help Desk and Medical Monitor contact information will be distributed to all investigators (see "Protocol Administrative and Contact Information & List of Investigators").

6.4 FOLLOW-UP OF PATIENTS AFTER ADVERSE EVENTS

6.4.1 Investigator Follow-Up

The investigator should follow each adverse event until the event has resolved to baseline grade or better, the event is assessed as stable by the investigator, the patient is lost to follow up, or the patient withdraws consent. Every effort should be made to follow all serious adverse events considered to be related to trial-related procedures until a final outcome can be reported.

During the study period, resolution of adverse events (with dates) should be documented on the Adverse Event eCRF and in the patient’s medical record to facilitate source data verification. If, after follow-up, return to baseline status or stabilization cannot be established, an explanation should be recorded on the Adverse Event eCRF.

7. STATISTICAL CONSIDERATIONS AND ANALYTICAL PLAN

RATIONALE FOR NUMBER OF PATIENTS

- The number of patients was selected based on clinical and experimental considerations.

- As Type I patients are particularly fragile and may not be able to provide sufficient samples for the proposed analyses, collections from this patient group for the purposes of this study are to be considered opportunistic. Collecting samples from less than 5 patients, or smaller volumes of blood samples, from Type I patients will still provide valuable qualitative information, and is therefore permitted.

7.1 SAFETY DATA ANALYSIS

7.1.1 Adverse Events

The original terms recorded on the patient's eCRF by the investigator for adverse events will be standardized by the sponsor by assigning preferred terms from the Medical Dictionary for Drug Regulatory Affairs (MedDRA).

Adverse events will be described by individual listings and frequency tables broken down by body system.

7.2 REPLACEMENT POLICY (ENSURING ADEQUATE NUMBERS OF EVALUABLE PATIENTS)

Patients may be replaced by additional patients if:

- Patient's specimen(s) collected are lost in transit
- Patient's specimen(s) are broken upon receipt
- Patient's specimen(s) are compromised or unanalyzable (i.e. hemolyzed) upon receipt
- Specimens of a particular type do not meet minimum volumes for processing
- No specimens of a particular type are collected during the visit
- Patient questionnaire information is not collected

Any clinical data collected or usable specimens for the patients replaced may be analyzed and used in association with the sample analysis.**ASSIGNMENT OF PREFERRED TERMS AND ORIGINAL TERMINOLOGY**

For classification purposes, preferred terms will be assigned by the sponsor to the original terms recorded on the eCRF, using the most up to date (as implemented at Roche) version of the Medical Dictionary for Regulatory Activities terminology (MedDRA) for AEs and diseases and the international non-proprietary name (INN) Drug Terms and Procedures Dictionary for treatments and surgical and medical procedures.

PART II: ETHICS AND GENERAL STUDY ADMINISTRATION

8. ETHICAL ASPECTS

8.1 LOCAL REGULATIONS/DECLARATION OF HELSINKI

The investigator will ensure that this study is conducted in full conformance with the principles of the “Declaration of Helsinki” or with the laws and regulations of the country in which the research is conducted, whichever affords the greater protection to the individual. The study must fully adhere to the principles outlined in current “Guideline for Good Clinical Practice” ICH Tripartite Guideline or with local law if it affords greater protection to the patient. For studies conducted in the EU/EEA countries, the investigator will ensure compliance with the current EU Clinical Trial Directive [2001/20/EC]. For studies conducted in the USA or under US IND, the investigator will additionally ensure adherence to the basic principles of “Good Clinical Practice” as outlined in the current version of 21 CFR, subchapter D, part 312, “Responsibilities of Sponsors and Investigators”, part 50, “Protection of Human Patients”, and part 56, “Institutional Review Boards”.

In other countries where “Guidelines for Good Clinical Practices” exist, Roche and the investigators will strictly ensure adherence to the stated provisions.

8.2 INFORMED CONSENT

Signed and dated IRB-approved informed consent must be obtained from the patient, or the patient’s parent or guardian before any study-specific screening procedures are performed. The Principal Investigator or approved designee will obtain informed consent from the parent or guardian and an informed assent from the pediatric patient (according to local institutional IRB guidelines) after full review of the pros and cons of the study. The possible benefits and complications of participation will be explained in detail. Every effort will be made to ensure that the patient and parent/guardian fully comprehend the nature of the study and the details of his or her participation. A copy of the consent form will be provided to the parent/guardian and patient. The informed consent process must be documented in the patient’s study record

8.3 INDEPENDENT ETHICS COMMITTEES/INSTITUTIONAL REVIEW BOARD

It is the understanding of the sponsor that this protocol (and any modifications) as well as appropriate consent procedures and advertisements, will be reviewed and approved by an Institutional Review Board (IRB). This board must operate in accordance with the current Federal Regulations. The sponsor will be sent a letter or certificate of approval prior to initiation of the study, and also whenever subsequent amendments /modifications are made to the protocol. Roche shall also submit an IND Annual Report to FDA according to local regulatory requirements and timelines.

9. CONDITIONS FOR MODIFYING THE PROTOCOL

Requests from investigators to modify the protocol to ongoing studies will be considered only after consultation between an appropriate representative of the sponsor and the investigator(s). Protocol modifications must be prepared by a representative of the sponsor.

All protocol modifications must be submitted to the appropriate IRB for information and/or approval in accordance with local requirements, and to Regulatory Agencies if required. Approval must be obtained before any changes can be implemented, except for changes necessary to eliminate an immediate hazard to study patients, or when the change(s) involves only logistical or administrative aspects of the study (e.g. change in monitor(s), change of telephone number(s).

10. CONDITIONS FOR TERMINATING THE STUDY

Both the sponsor and the investigator reserve the right to terminate the study at any time. Should this be necessary, both parties will arrange the procedures on an individual study basis after review and consultation. In terminating the study, Roche and the investigator will assure that adequate consideration is given to the protection of the patient's interests. The appropriate IRB and Regulatory Agencies should be informed accordingly.

11. STUDY DOCUMENTATION, CRF OR ECRF AND RECORD KEEPING

11.1 INVESTIGATOR'S FILES/RETENTION OF DOCUMENTS

The Investigator must maintain adequate and accurate records to enable the conduct of the study to be fully documented and the study data to be subsequently verified. These documents should be classified into two different separate categories (1) Investigator's Study File, and (2) patient clinical source documents.

The Investigator's Study File will contain the protocol/amendments, eCRF data and Discrepancies, IRB and regulatory authority approval with correspondence, sample informed consent, , staff curriculum vitae and authorization forms and other appropriate documents/correspondence, etc.

Patient clinical source documents (usually defined by the project in advance to record key efficacy/safety parameters independent of the eCRF) would include patient hospital/clinic records, physician's and nurse's notes, original laboratory reports, ECG, EEG, X–ray, pathology and special assessment reports, signed informed consent forms, consultant letters, and patient screening and enrollment logs. The Investigator must keep these two categories of documents on file for at least 15 years after completion or discontinuation of the study. After that period of time the investigator will notify Roche that the documents will be destroyed, patient to local regulations.

Should the Investigator wish to assign the study records to another party or move them to another location, Roche must be notified in advance.

If the Investigator can not guarantee this archiving requirement at the investigational site for any or all of the documents, special arrangements must be made between the Investigator and Roche to store these in a sealed container(s) outside of the site so that they can be returned sealed to the Investigator in case of a regulatory audit. Where source documents are required for the continued care of the patient, appropriate copies should be made for storing outside of the site.

11.2 SOURCE DOCUMENTS AND BACKGROUND DATA

The investigator shall supply the sponsor on request with any required background data from the study documentation or clinic records. This is particularly important when errors in data transcription are suspected. In case of special problems and/or governmental queries or requests for audit inspections, it is also necessary to have access to the complete study records, provided that patient confidentiality is protected.

11.3 AUDITS AND INSPECTIONS

The investigator should understand that source documents for this study should be made available to appropriately qualified personnel from the Roche Pharma Development Quality Assurance Unit or its designees, or to health authority inspectors after appropriate notification. The verification of the eCRF data must be by direct inspection of source documents.

11.4 ELECTRONIC CASE REPORT FORMS

Data for this study will be captured via an on line Electronic Data Capture (EDC) system. The data collected in the source documents is entered onto the study eCRF. An audit trail will maintain a record of initial entries and changes made; reasons for change; time and date of entry; and user name of person authorizing entry or change. For each patient enrolled, an eCRF must be completed and electronically signed by the principal investigator or authorized delegate from the study staff. If a patient withdraws from the study, the reason must be noted on the eCRF. The investigator should ensure the accuracy, completeness and timeliness of the data reported to the sponsor in the eCRFs and in all required reports.

12. MONITORING THE STUDY

It is understood that the responsible Roche monitor (or designee) will contact and visit the investigator regularly and will be allowed, on request, to inspect the various records of the study (eCRF and other pertinent data) provided that patient confidentiality is maintained in accordance with local requirements.

It will be the monitor's responsibility to inspect the eCRF at regular intervals throughout the study, to verify the adherence to the protocol and the completeness, consistency and

accuracy of the data being entered on them.. The monitor should have access to laboratory test reports and other patient records needed to verify the entries on the eCRF. The investigator (or deputy) agrees to cooperate with the monitor to ensure that any problems detected in the course of these monitoring visits are resolved.

13. CONFIDENTIALITY OF TRIAL DOCUMENTS AND PATIENT RECORDS

The investigator must assure that patients' anonymity will be maintained and that their identities are protected from unauthorized parties. On eCRF or other documents submitted to the sponsor, patients should not be identified by their names, but by an identification code. The investigator should keep a patient enrollment log showing codes patient names and addresses. The investigator should maintain documents not for submission to Roche, e.g., patients' written consent forms, in strict confidence.

Roche already maintains rigorous confidentiality standards for clinical studies by "coding" (i.e. assigning a unique patient ID number at the investigator site) all patients enrolled in Roche clinical studies. This means that patient names are not included in data sets that are transmitted to any Roche location. Given the sensitive nature of genetic data, Roche has implemented a number of additional processes to assure patient confidentiality Publication Of Data And Protection Of Trade Secrets

Roche will comply with the requirements for publication of study results.

The results of this study may be published or presented at scientific meetings. If this is foreseen, the investigator agrees to submit all manuscripts or abstracts to Roche prior to submission. This allows the sponsor to protect proprietary information and to provide comments based on information from other studies that may not yet be available to the investigator.

In accordance with standard editorial and ethical practice, Roche will generally support publication of multi-center trials only in their entirety and not as individual center data. In this case, a coordinating investigator will be designated by mutual agreement.

Any formal publication of the study in which input of Roche personnel exceeded that of conventional monitoring will be considered as a joint publication by the investigator and the appropriate Roche personnel.
