# Supplementary material for: Biomarker for Spinal Muscular Atrophy: Expression of SMN in Peripheral Blood of SMA Patients and Healthy Controls
Source: PLoS One. 2015 Oct 15;10(10):e0139950. doi: 10.1371/journal.pone.0139950 (PMC4607439; doi:10.1371/journal.pone.0139950)
Supplement: S1 Table — All data and values from the different assays of SMA patients and Healthy controls (HC). Abbreviations are: SMN1FL = SMN1 full length, SMN2FL = SMN2 full length SMN2d7 = SMN delta Exon 7, SMN-RG = Reference Gene used for the SMN Cobas assay. CR = Relative expression (also known as Concentration Ratio, CR) is calculated using 2^-deltaCp, where deltaCp = Target Cp − Reference Cp. (PDF) [file pone.0139950.s004.pdf]

					Roche Cobas RT-PCR assay														
Study	Sample ID	Donor ID	SMN2 as published in (13)	GAPDH using Taqman No 4310884E	SMN1FL Cp1	SMN2FL Cp2	SMN7D Cp3	SMN-RG Cp4	deltaCp SMN1FL	deltaCp SMN2FL	deltaCp SMN7D	CR with SMN1FL	CR with SMN2FL	CR with SMN7D	SMN1	SMN2	Type	SMN protein pg/ml	AGE in years
BE29002		2020			nd	24.21	24.38	24.04	nd	0.17	0.34		0.89	0.79	0	3	2	2533	7
BE29002		2019			nd	24.26	24.56	24.35	nd	-0.09	0.21		1.06	0.86	0	3	3	3250	2
BE29002		2018			nd	24.65	24.82	24.3	nd	0.35	0.52		0.78	0.70	0	3	2	2208	12
BE29002		2017			nd	23.89	24.24	23.98	nd	-0.09	0.26		1.06	0.84	0	3	2	3931	0
BE29002		2016			nd	24.7	25.11	24.3	nd	0.4	0.81		0.76	0.57	0	3	3	2612	45
BE29002		2015			nd	24.62	25.09	24.22	nd	0.4	0.87		0.76	0.55	0	3	3	2298	45
BE29002		2014			nd	24.62	24.81	24.3	nd	0.32	0.51		0.80	0.70			1	3327	0
BE29002		2013			nd	25.6	26.03	25.45	nd	0.15	0.58		0.90	0.67	0	4	3	2608	6
BE29002		2012			nd	24.16	24.54	24.28	nd	-0.12	0.26		1.09	0.84	0	4	3	3444	4
BE29002		2011			nd	25.17	25.48	24.42	nd	0.75	1.06		0.59	0.48	0	2	1	1307	9
BE29002		2010			nd	24.59	24.78	24.08	nd	0.51	0.7		0.70	0.62	0	3	2	1959	57
BE29002		2009			nd	24.96	25.1	25.03	nd	-0.07	0.07		1.05	0.95	0	4	3	3483	13
BE29002		2008			nd	24.98	25.01	24.92	nd	0.06	0.09		0.96	0.94	0	4	3	4273	10
BE29002		2007			nd	25.29	25.35	24.57	nd	0.72	0.78		0.61	0.58	0	2	1	1759	5
BE29002		2006			nd	24.27	24.51	24.04	nd	0.23	0.47		0.85	0.72	0	3	2	3846	2
BE29002		2005			nd	24.47	24.69	24.52	nd	-0.05	0.17		1.04	0.89	0	3	3	2151	3
BE29002		2004			nd	24.57	24.86	24.15	nd	0.42	0.71		0.75	0.61	0	2	1	2858	0
BE29002		2003			nd	24.49	24.78	24.5	nd	-0.01	0.28		1.01	0.82	0	3	3	2585	2
BE29002		2002			nd	24.42	24.61	24.26	nd	0.16	0.35		0.90	0.78	0	3	2	2101	3
BE29002		2001			nd	24.38	24.5	24.09	nd	0.29	0.41		0.82	0.75	0	2	1	3886	0
BE29002		1016			nd	25.69	25.63	24.68	nd	1.01	0.95		0.50	0.52	0	2	1	1569	2
BE29002		1015			nd	26.11	26.35	25.79	nd	0.32	0.56		0.80	0.68	0	3	2	2489	16
BE29002		1014			nd	25.5	25.6	25.05	nd	0.45	0.55		0.73	0.68	0	3	2	2058	14
BE29002		1013			nd	25.6	25.83	25.39	nd	0.21	0.44		0.86	0.74	0	4	3	4287	21
BE29002		1012			nd	25.32	25.57	24.62	nd	0.7	0.95		0.62	0.52	0	3	3	2504	26
BE29002		1011			nd	24.6	24.76	24.17	nd	0.43	0.59		0.74	0.66	0	4	3	3248	46
BE29002		1010			nd	25.29	25.52	24.67	nd	0.62	0.85		0.65	0.55	0	3	2	2441	9
BE29002		1009			nd	25.32	25.53	24.51	nd	0.81	1.02		0.57	0.49	0	3	3	3190	8
BE29002		1008			nd	24.5	24.59	23.97	nd	0.53	0.62		0.69	0.65	0	2	1	3007	0
BE29002		1007			nd	25.03	25.01	24.63	nd	0.4	0.38		0.76	0.77	0	3	2	3541	3
BE29002		1006			nd	25.01	25.25	24.75	nd	0.26	0.5		0.84	0.71	0	3	2	4288	9
BE29002		1005			nd	25.08	25.13	24.48	nd	0.6	0.65		0.66	0.64	0	3	2	2105	4
BE29002		1004			nd	24.31	24.63	24.23	nd	0.08	0.4		0.95	0.76	0	3	2	3351	6
BE29002		1003			nd	23.94	24.02	23.77	nd	0.17	0.25		0.89	0.84	0	4	3	3505	22
BE29002		1002			nd	25.37	25.92	24.79	nd	0.58	1.13		0.67	0.46	0	4	3	4362	61
BE29002		1001			nd	24.6	24.79	24.51	nd	0.09	0.28		0.94	0.82	0	4	2	4185	8
HC-Bloodbank	96	561	27.2	21.6	25.61	27.01	24.86	23.88	1.73	3.13	0.98	0.30	0.11	0.51	2	2	HC	nd	
HC-Bloodbank	95	560	27.6	21.7	26.21	28.29	26.1	24.93	1.28	3.36	1.17	0.41	0.10	0.44	2	1	HC	nd	
HC-Bloodbank	94	559	25.7	21.1	24.79	25.86	23.68	23.28	1.51	2.58	0.4	0.35	0.17	0.76	2	2	HC	nd	
HC-Bloodbank	93	557	25.5	25.1	24.51	25.63	23.47	22.93	1.58	2.7	0.54	0.33	0.15	0.69	2	2	HC	nd	
HC-Bloodbank	92	555	26.7	21.9	26.08	27.28	25.01	24.45	1.63	2.83	0.56	0.32	0.14	0.68	2	2	HC	nd	
HC-Bloodbank	91	554	28.0	22.5	24.32	25.55	23.45	23.04	1.28	2.51	0.41	0.41	0.18	0.75	2	2	HC	nd	
HC-Bloodbank	90	551	30.7	22.5	25.54	nd	nd	24.64	0.9	nd	nd	0.54	0.00	nd	3	0	HC	nd	
HC-Bloodbank	89	550	27.8	22.7	24.69	26.93	24.61	23.22	1.47	3.71	1.39	0.36	0.08	0.38	2	1	HC	nd	
HC-Bloodbank	88	548	28.8	22.8	24.75	25.92	23.74	23.19	1.56	2.73	0.55	0.34	0.15	0.68	2	2	HC	nd	
HC-Bloodbank	87	547	27.5	20.9	24.9	26.07	23.93	23.32	1.58	2.75	0.61	0.33	0.15	0.66	2	2	HC	nd	
HC-Bloodbank	86	546	27.0	20.5	24.03	25.64	23.39	22.74	1.29	2.9	0.65	0.41	0.13	0.64	3	2	HC	nd	
HC-Bloodbank	85	545	28.5	21.6	25.77	27.95	25.35	24.08	1.69	3.87	1.27	0.31	0.07	0.41	2	1	HC	nd	
HC-Bloodbank	84	543	27.9	21.6	26.79	29.15	26.48	25.27	1.52	3.88	1.21	0.35	0.07	0.43	2	1	HC	nd	
HC-Bloodbank	83	537	28.8	22.0	26.33	28.77	26.19	24.74	1.59	4.03	1.45	0.33	0.06	0.37	2	1	HC	nd	
HC-Bloodbank	82	535	27.4	20.7	25.74	27.05	25	24.6	1.14	2.45	0.4	0.45	0.18	0.76	2	2	HC	nd	

HC-Bloodbank	81	533	27.2	20.1	23.7	26.16	23.6	22.22	1.48	3.94	1.38	0.36	0.07	0.38	2	1	HC	nd
HC-Bloodbank	80	531	28.4	21.7	24.51	25.68	23.26	22.76	1.75	2.92	0.5	0.30	0.13	0.71	2	2	HC	nd
HC-Bloodbank	79	529	28.8	22.7	22.87	26.33	22.72	21.68	1.19	4.65	1.04	0.44	0.04	0.49	3	1	HC	nd
HC-Bloodbank	78	527	27.0	20.7	25.08	26.34	24.15	23.3	1.78	3.04	0.85	0.29	0.12	0.55	2	2	HC	nd
HC-Bloodbank	77	526	27.0	20.5	25.28	26.56	24.36	23.65	1.63	2.91	0.71	0.32	0.13	0.61	2	2	HC	nd
HC-Bloodbank	76	524	28.0	21.4	26.07	29.17	26.36	24.99	1.08	4.18	1.37	0.47	0.06	0.39	2	2	HC	nd
HC-Bloodbank	75	523	26.9	21.9	25.17	26.48	24.22	23.7	1.47	2.78	0.52	0.36	0.15	0.70	3	1	HC	nd
HC-Bloodbank	74	521	27.6	20.5	24.26	25.63	23.25	22.9	1.36	2.73	0.35	0.39	0.15	0.78	2	2	HC	nd
HC-Bloodbank	73	520	28.4	21.1	nd	nd	nd	nd	nd	nd	nd	nd	nd	nd	2	1	HC	nd
HC-Bloodbank	72	519	27.8	22.7	25.8	26.85	24.57	23.97	1.83	2.88	0.6	0.28	0.14	0.66	2	2	HC	nd
HC-Bloodbank	71	518	26.1	21.3	25.34	26.41	24.03	23.56	1.78	2.85	0.47	0.29	0.14	0.72	2	2	HC	nd
HC-Bloodbank	70	517	27.7	20.2	25.75	28	25.31	24.1	1.65	3.9	1.21	0.32	0.07	0.43	2	1	HC	nd
HC-Bloodbank	69	516	27.4	20.6	24.81	26.07	23.67	23.27	1.54	2.8	0.4	0.34	0.14	0.76	2	2	HC	nd
HC-Bloodbank	68	512	28.5	21.4	26.64	28.92	26.47	25.07	1.57	3.85	1.4	0.34	0.07	0.38	2	1	HC	nd
HC-Bloodbank	67	508	27.0	20.6	25.57	26.76	24.71	23.97	1.6	2.79	0.74	0.33	0.14	0.60	2	2	HC	nd
HC-Bloodbank	66	506	28.1	20.3	24.9	27.33	24.6	23.31	1.59	4.02	1.29	0.33	0.06	0.41	2	1	HC	nd
HC-Bloodbank	65	505	28.2	21.4	24.6	27.06	24.17	22.88	1.72	4.18	1.29	0.30	0.06	0.41	2	1	HC	nd
HC-Bloodbank	64	503	27.4	14.0	24.95	27.22	24.55	23.01	1.94	4.21	1.54	0.26	0.05	0.34	2	1	HC	nd
HC-Bloodbank	63	502	28.4	20.7	27.17	29.38	26.43	24.21	2.96	5.17	2.22	0.13	0.03	0.21	2	1	HC	nd
HC-Bloodbank	62	500	28.0	20.8	25.95	27.07	24.79	24.01	1.94	3.06	0.78	0.26	0.12	0.58	2	2	HC	nd
HC-Bloodbank	61	499	27.7	20.9	25.74	26.91	24.57	23.95	1.79	2.96	0.62	0.29	0.13	0.65	2	2	HC	nd
HC-Bloodbank	60	498	28.2	21.4	25.34	27.41	25.09	23.67	1.67	3.74	1.42	0.31	0.07	0.37	2	1	HC	nd
HC-Bloodbank	59	497	28.3	20.8	26.16	28.45	25.98	24.61	1.55	3.84	1.37	0.34	0.07	0.39	2	1	HC	nd
HC-Bloodbank	58	493	27.2	20.5	24.4	25.62	23.28	23	1.4	2.62	0.28	0.38	0.16	0.82	2	2	HC	nd
HC-Bloodbank	57	492	27.4	20.6	24.25	25.43	23.2	22.63	1.62	2.8	0.57	0.33	0.14	0.67	2	2	HC	nd
HC-Bloodbank	56	491	28.5	14.9	24.94	26.71	24.12	23.66	1.28	3.05	0.46	0.41	0.12	0.73	3	2	HC	nd
HC-Bloodbank	55	490	28.2	26.6	26.26	28.42	25.71	24.33	1.93	4.09	1.38	0.26	0.06	0.38	2	1	HC	nd
HC-Bloodbank	54	487	27.8	21.3	24.5	26.78	23.87	22.61	1.89	4.17	1.26	0.27	0.06	0.42	2	1	HC	nd
HC-Bloodbank	53	486	27.1	20.8	24.91	26	23.6	23.04	1.87	2.96	0.56	0.27	0.13	0.68	2	2	HC	nd
HC-Bloodbank	52	483	27.8	21.1	26.58	29.07	26.16	25.03	1.55	4.04	1.13	0.34	0.06	0.46	2	1	HC	nd
HC-Bloodbank	51	482	27.9	20.2	24.89	27.18	24.6	23.35	1.54	3.83	1.25	0.34	0.07	0.42	2	1	HC	nd
HC-Bloodbank	50	481	28.4	20.3	25.28	27.55	25.07	23.56	1.72	3.99	1.51	0.30	0.06	0.35	2	1	HC	nd
HC-Bloodbank	49	480	27.8	21.0	25.42	27.91	25.11	23.73	1.69	4.18	1.38	0.31	0.06	0.38	2	1	HC	nd
HC-Bloodbank	48	479	27.9	22.0	24.73	25.82	23.62	23.12	1.61	2.7	0.5	0.33	0.15	0.71	2	2	HC	nd
HC-Bloodbank	47	478	28.4	20.9	24.93	27.13	24.65	23.27	1.66	3.86	1.38	0.32	0.07	0.38	2	1	HC	nd
HC-Bloodbank	46	474	31.4	22.0	25.87	nd	nd	24.38	1.49	nd	nd	0.36		nd	2	0	HC	nd
HC-Bloodbank	45	473	27.9	20.8	25.05	27.59	25.34	23.94	1.11	3.65	1.4	0.46	0.08	0.38	3	1	HC	nd
HC-Bloodbank	44	472	28.3	20.9	25.59	27.82	25.43	24.12	1.47	3.7	1.31	0.36	0.08	0.40	2	1	HC	nd
HC-Bloodbank	43	471	27.4	20.6	25.37	26.54	24.46	23.73	1.64	2.81	0.73	0.32	0.14	0.60	2	2	HC	nd
HC-Bloodbank	42	470	27.0	20.1	25.76	26.82	24.83	24.24	1.52	2.58	0.59	0.35	0.17	0.66	2	2	HC	nd
HC-Bloodbank	41	468	26.4	19.8	25.1	26.36	24.17	23.55	1.55	2.81	0.62	0.34	0.14	0.65	2	2	HC	nd
HC-Bloodbank	40	467	28.2	21.6	26.15	28.41	25.92	24.23	1.92	4.18	1.69	0.26	0.06	0.31	2	2	HC	nd
HC-Bloodbank	39	466	27.6	14.5	26.24	27.39	25.05	24.5	1.74	2.89	0.55	0.30	0.13	0.68	2	2	HC	nd
HC-Bloodbank	38	465	27.2	26.2	24.41	25.47	23.16	22.61	1.8	2.86	0.55	0.29	0.14	0.68	2	1	HC	nd
HC-Bloodbank	37	464	28.3	21.8	24.36	26.49	24.07	22.7	1.66	3.79	1.37	0.32	0.07	0.39	2	2	HC	nd
HC-Bloodbank	36	461	27.5	21.1	24.06	25.18	23.15	22.58	1.48	2.6	0.57	0.36	0.16	0.67	2	1	HC	nd
HC-Bloodbank	35	460	27.0	20.9	26.34	27.53	25.28	24.57	1.77	2.96	0.71	0.29	0.13	0.61	2	2	HC	nd
HC-Bloodbank	34	459	27.8	22.3	26.02	27.2	25.06	24.18	1.84	3.02	0.88	0.28	0.12	0.54	2	2	HC	nd
HC-Bloodbank	33	457	28.2	21.9	25.82	28.12	25.74	24.23	1.59	3.89	1.51	0.33	0.07	0.35	2	1	HC	nd
HC-Bloodbank	32	455	28.2	21.7	26.55	28.69	25.94	24.46	2.09	4.23	1.48	0.23	0.05	0.36	2	1	HC	nd
HC-Bloodbank	31	453	28.2	13.9	26.28	28.47	25.99	24.33	1.95	4.14	1.66	0.26	0.06	0.32	2	1	HC	nd

HC-Bloodbank	30	452	28.1	21.5	24.47	26.55	24.2	22.92	1.55	3.63	1.28	0.34	0.08	0.41	2	1	HC	nd
HC-Bloodbank	29	451	26.7	21.3	25.52	26.75	24.45	23.89	1.63	2.86	0.56	0.32	0.14	0.68	2	2	HC	nd
HC-Bloodbank	28	450	27.8	20.8	24.68	26.93	24.47	23.14	1.54	3.79	1.33	0.34	0.07	0.40	2	1	HC	nd
HC-Bloodbank	27	448	27.6	14.9	26.71	27.89	25.64	24.97	1.74	2.92	0.67	0.30	0.13	0.63	2	2	HC	nd
HC-Bloodbank	26	447	28.1	20.7	25.72	28.08	25.43	24.25	1.47	3.83	1.18	0.36	0.07	0.44	2	1	HC	nd
HC-Bloodbank	25	446	28.3	22.4	25.74	27.11	24.72	23.91	1.83	3.2	0.81	0.28	0.11	0.57	2	2	HC	nd
HC-Bloodbank	24	445	27.3	20.8	25.65	26.82	24.27	23.67	1.98	3.15	0.6	0.25	0.11	0.66	2	2	HC	nd
HC-Bloodbank	23	444	27.4	26.1	26.88	28.88	26.39	25.82	1.06	3.06	0.57	0.48	0.12	0.67	4	2	HC	nd
HC-Bloodbank	22	443	27.5	20.6	27.01	28.1	25.73	25.01	2	3.09	0.72	0.25	0.12	0.61	2	2	HC	nd
HC-Bloodbank	21	442	26.9	20.3	24.92	26.22	23.64	22.76	2.16	3.46	0.88	0.22	0.09	0.54	2	2	HC	nd
HC-Bloodbank	20	439	27.7	20.9	25.87	27.05	24.86	23.93	1.94	3.12	0.93	0.26	0.12	0.52	2	2	HC	nd
HC-Bloodbank	19	438	27.7	20.8	25.29	26.81	24.14	23.19	2.1	3.62	0.95	0.23	0.08	0.52	2	2	HC	nd
HC-Bloodbank	18	437	26.9	20.0	25.56	26.79	24.47	23.66	1.9	3.13	0.81	0.27	0.11	0.57	2	2	HC	nd
HC-Bloodbank	17	436	27.7	20.7	23.16	26.21	23.32	21.96	1.2	4.25	1.36	0.44	0.05	0.39	3	1	HC	nd
HC-Bloodbank	16	435	27.8	21.1	25.69	26.82	24.4	23.82	1.87	3	0.58	0.27	0.13	0.67	2	2	HC	nd
HC-Bloodbank	15	434	28.3	21.6	25.64	27.66	25.17	23.87	1.77	3.79	1.3	0.29	0.07	0.41	2	1	HC	nd
HC-Bloodbank	14	433	27.6	20.2	26.17	27.23	24.92	24.04	2.13	3.19	0.88	0.23	0.11	0.54	2	2	HC	nd
HC-Bloodbank	13	432	26.8	20.3	25.68	27.17	24.68	23.92	1.76	3.25	0.76	0.30	0.11	0.59	2	2	HC	nd
HC-Bloodbank	12	431	27.7	20.7	26.52	27.68	25.36	24.68	1.84	3	0.68	0.28	0.13	0.62	2	2	HC	nd
HC-Bloodbank	11	430	27.8	26.3	25.57	27.81	25.15	23.69	1.88	4.12	1.46	0.27	0.06	0.36	2	1	HC	nd
HC-Bloodbank	10	427	27.6	22.0	25.08	26.58	24.11	23.36	1.72	3.22	0.75	0.30	0.11	0.59	2	2	HC	nd
HC-Bloodbank	9	423	29.3	27.7	25.19	27.53	24.88	23.37	1.82	4.16	1.51	0.28	0.06	0.35	2	1	HC	nd
HC-Bloodbank	8	422	26.8	21.6	25.4	26.43	23.82	23.36	2.04	3.07	0.46	0.24	0.12	0.73	2	2	HC	nd
HC-Bloodbank	7	421	30.2	20.4	25.86	nd	nd	23.52	2.34	nd	nd	0.20		nd	2	0	HC	nd
HC-Bloodbank	6	420	26.7	25.7	25.98	27.05	24.79	23.93	2.05	3.12	0.86	0.24	0.12	0.55	2	2	HC	nd
HC-Bloodbank	5	418	27.1	20.6	25.09	26.24	24.11	23.34	1.75	2.9	0.77	0.30	0.13	0.59	2	2	HC	nd
HC-Bloodbank	4	417	28.2	21.0	24.67	26.86	24.34	23.14	1.53	3.72	1.2	0.35	0.08	0.44	2	1	HC	nd
HC-Bloodbank	3	416	26.9	21.8	nd	nd	nd	nd	nd	nd	nd	nd	nd	nd	2	2	HC	nd
HC-Bloodbank	2	415	30.7	27.2	26.91	nd	nd	24.78	2.13	nd	nd	0.23	0.00	nd	2	0	HC	nd
HC-Bloodbank	1	414	28.3	20.6	24.52	27.02	24.23	22.55	1.97	4.47	1.68	0.26	0.05	0.31	2	1	HC	nd

13. Naryshkin NA, Weetall M, Dakka A, Narasimhan J, Zhao X, et al. (2014) Motor neuron disease. SMN2 splicing modifiers improve motor function and longevity in mice with spinal muscular atrophy. Science 345: 688-693
"nd= "not detectable"
